# A Systematic Review and Meta‐Analysis of Malnutrition and Metabolic Failure in High‐Potency Incretin Therapy

**DOI:** 10.1002/osp4.70188

**Published:** 2026-09-06

**Authors:** Eugene Ampofo, Charles Apprey, Mary Amoako, Fiifi Derrick Turkson

**Affiliations:** ^1^ Department of Biochemistry and Biotechnology Kwame Nkrumah University of Science and Technology University Post Office Kumasi Ghana; ^2^ Department of Economics Kwame Nkrumah University of Science and Technology University Post Office Kumasi Ghana

**Keywords:** malnutrition, sarcopenic Obesity, semaglutide, subclinical malnutrition, tirzepatide, treatment‐emergent adverse events

## Abstract

**Objective:**

High‐potency incretin therapy achieved substantial weight loss, but the extreme energy deficits it induced may obscure the underlying nutritional deterioration. This meta‐analysis synthesized data from 19 randomized trials across the SURMOUNT, STEP, SCALE, and OASIS programs to quantify the nutritional and body composition consequences of these agents in adults with obesity.

**Methods:**

This systematic review and meta‐analysis followed PRISMA 2020 guidelines. Risk of bias was assessed using the Cochrane RoB2 tool, and certainty of evidence was rated using the GRADE approach. Continuous outcomes were pooled using mean differences within a random‐effects model.

**Results:**

Daily energy intake declined by 24.00%–39.20% across drug classes, with model‐estimated daily deficits reaching 1200 kcal. Tirzepatide 15 mg was associated with a mean fat‐free mass (FFM) reduction of 1.60 kg, representing 2.80% of body weight. Investigator‐reported malnutrition occurred in only 0.12% of participants. Objective laboratory screening identified low total lymphocyte counts below 910 per microliter in 2.90% of active‐therapy participants, nearly double the 1.77% in placebo arms, indicating that standard adverse event reporting underestimates true nutritional risk. Pancreatic lipase increased by a mean of 31% in the SCALE program, representing a secondary metabolic signal.

**Conclusions:**

Given these findings, a Tiered Stepped‐Care Algorithm is proposed, mandating baseline screening of albumin and total lymphocyte count, periodic monitoring at weeks 12, 24, and 52, and defined intervention thresholds to prevent sarcopenia and frailty, particularly in adults aged 65 years and older.

## 1 Introduction

Current Phase 3 trial programmes, including SURMOUNT [1, 2, 3], STEP [4, 5, 6, 7, 8, 9], SCALE [10, 11, 12, 13], and OASIS [14], were designed primarily to demonstrate weight loss efficacy and cardiovascular or metabolic safety. Systematic, pre‐specified nutritional monitoring was largely absent from these protocols [15]. Consequently, investigator‐reported malnutrition events appeared rare. However, objective biomarkers tell a different story. Low total lymphocyte counts, a sensitive index of protein‐energy status, were detected in 2.90% of participants on active therapy compared with 1.77% in placebo arms [15]. This discrepancy suggests that clinically meaningful nutritional deterioration occurred beneath the threshold of standard adverse event reporting.

The clinical adoption of these therapies expanded rapidly after regulatory approval, driven by consistent evidence of weight loss efficacy across trial programmes. A large‐scale study of real‐world outcomes using United States Department of Veterans Affairs data documented a broad and expanding risk profile linked to GLP‐1 receptor agonist use across different patient groups, including increased risk of gastrointestinal disorders and drug‐induced pancreatitis alongside reduced risk of several cardiometabolic and neurocognitive outcomes [16]. This rapid growth in use outpaced the nutritional monitoring systems that had long been the standard for bariatric surgery patients. As a result, a large population was exposed to fast, drug‐induced weight loss without a matching standard of nutritional oversight.

A growing body of independent research supports this concern. A 2024 review in Circulation reported that lean mass losses ranged from about 25% to 40% of total weight lost with semaglutide, and up to 60% with liraglutide. These figures were broadly similar to those seen after bariatric surgery [17]. A later systematic review and meta‐analysis in Obesity Reviews, covering people with and without type 2 diabetes, confirmed consistent reductions in lean mass across different agents and doses [18]. These findings suggest that lean mass loss was not a rare signal buried in secondary analyses. It was a repeated feature of high‐potency incretin therapy that needed a systematic review across the full body of trials.

Beyond lean mass and protein status, the hedonic blunting induced by these agents may have reduced spontaneous dietary variety, potentially compromising intake of fiber, iron, vitamin D, and vitamin B12 at a time when micronutrient needs remained unchanged or increased [4, 19]. Secondary physiological stressors also warrant attention. The SCALE program documented a 31% mean increase in pancreatic lipase, raising questions about subclinical pancreatic stress and its potential contribution to fat‐soluble vitamin malabsorption [10]. Rapid weight reduction also has implications for bone mineral density. Obesity increased bone mass through mechanical loading. When weight was lost rapidly, this loading stimulus was removed. The result may have been accelerated bone resorption, particularly in postmenopausal women and older adults [20, 21].

This meta‐analysis addressed these gaps by quantifying the magnitude of treatment‐induced caloric restriction, lean mass attrition, and subclinical nutritional risk signals across 19 high‐potency trials. A stratified synthesis was presented, acknowledging that liraglutide 3.0 mg, oral semaglutide 50 mg, and trials conducted in populations with type 2 diabetes represented distinct clinical contexts.

## 2 Methods

PRISMA 2020 standards guided the conduct and reporting of this meta‐analysis [22].

### 2.1 Study Eligibility and PICOTS Framework

For the purposes of this review, high‐potency incretin therapy was defined as agents or doses associated with ≥ 10% mean total body weight reduction in Phase 3 randomised controlled trials, a threshold met by tirzepatide (all doses) and semaglutide ≥ 1.0 mg (injectable) and semaglutide 50 mg (oral). Liraglutide 3.0 mg did not consistently meet this threshold. It was therefore classified as a moderate‐potency comparator rather than a high‐potency agent. Its inclusion was retained because the SCALE program provided the only available systematic measurements of pancreatic enzyme changes across the incretin class. Two included trials enrolled participants with type 2 diabetes: SURPASS‐AP‐Combo [23] and STEP 2 [5]. These were retained because the primary outcomes, namely energy intake suppression and fat‐free mass change, reflected pharmacological mechanisms independent of baseline glycemic status. Sensitivity analyses excluding these trials did not materially alter the pooled estimates. Adults aged ≥ 18 years with a BMI ≥ 30 kg/m^2^ or ≥ 27 kg/m^2^ and at least one weight‐related complication were eligible. Control arms utilized placebo or active comparators, and a minimum trial duration of 12 weeks was required to capture the metabolic shift from the initial rapid weight loss into the physiological maintenance phase.

### 2.2 Information Sources and Search Strategy

The systematic search was conducted across PubMed, Scopus, and Web of Science. A focused search string was applied to target the intersection of drug potency and metabolic failure: ((“*tirzepatide*” OR “*semaglutide*” OR “*liraglutide 3.0 mg*”) AND (“*malnutrition*” OR “*energy intake*” OR “*caloric restriction*” OR “*body composition*” OR “*fat‐free mass*” OR “*sarcopenia*” OR “*albumin*” OR “*total lymphocyte count*”) AND (“*randomized controlled trial*” OR “*post‐hoc analysis*”)). Reference lists from the retrieved reviews provided additional primary data sources. The initial systematic search was conducted on February 12, 2026, across all three databases with no lower date limit. To ensure literature coverage through July 2026, an updated search using the same search string was conducted on July 31, 2026, identifying seven additional records, none of which met the inclusion criteria at title and abstract screening.

### 2.3 Selection Process and Attrition Logic

The initial search identified 871 potential sources; an updated search extending coverage through July 2026 identified 7 additional records, for a combined total of 878. Removing 386 duplicates left 492 records for screening. Independent reviewers excluded 420 items for failing to meet drug potency, study design (not a randomized controlled trial), or 12‐week duration thresholds. Of the 72 reports undergoing full‐text evaluation, 53 were excluded because they lacked standardized DXA‐based body composition or specific nutritional laboratory markers such as total lymphocyte count. Nineteen high‐potency trials met all the criteria and provided the data for the final synthesis.

### 2.4 Outcome Measures and Data Extraction

The primary outcome was the pooled incidence of MedDRA‐coded malnutrition events, such as abnormal weight loss and hypoalbuminemia. Secondary measures quantified the physiological mechanisms of treatment‐induced nutritional risk: percentage reductions in *ad libitum* energy intake and absolute loss of fat‐free mass (FFM) in kilograms. Scaling discrepancies in energy reporting were converted into Megajoules (MJ) for statistical pooling. Subclinical risk signals, specifically albumin < 3.3 g/dL and total lymphocyte count (TLC) < 910/μL, were extracted alongside secondary metabolic stressors like mean lipase increases.

### 2.5 Quality Assessment and Risk of Bias

The Cochrane Risk‐of‐Bias tool (RoB2) was applied to all randomized trials. Domains evaluated included randomization integrity, deviations from interventions, and missing outcome data. Safety data were drawn from modified intention‐to‐treat populations to maintain conservative estimates of malnutrition prevalence.

### 2.6 Statistical Synthesis and Subgroup Modeling

Continuous variables, including energy intake and muscle mass loss were analyzed using mean differences (MD) within a random‐effects model. A pre‐specified subgroup analysis was conducted for adults aged 65 years and older to determine whether drug‐induced FFM loss compounded age‐related sarcopenia. Statistical heterogeneity was quantified using the *I* ^2^ statistic in RevMan software.

### 2.7 Certainty Assessment

The certainty of the evidence for each primary and secondary outcome followed the GRADE (Grading of Recommendations Assessment, Development and Evaluation) approach. This assessment weighed the risk of bias, inconsistency, and imprecision to determine the certainty of evidence for each outcome.

## 3 Results

**FIGURE 1:**
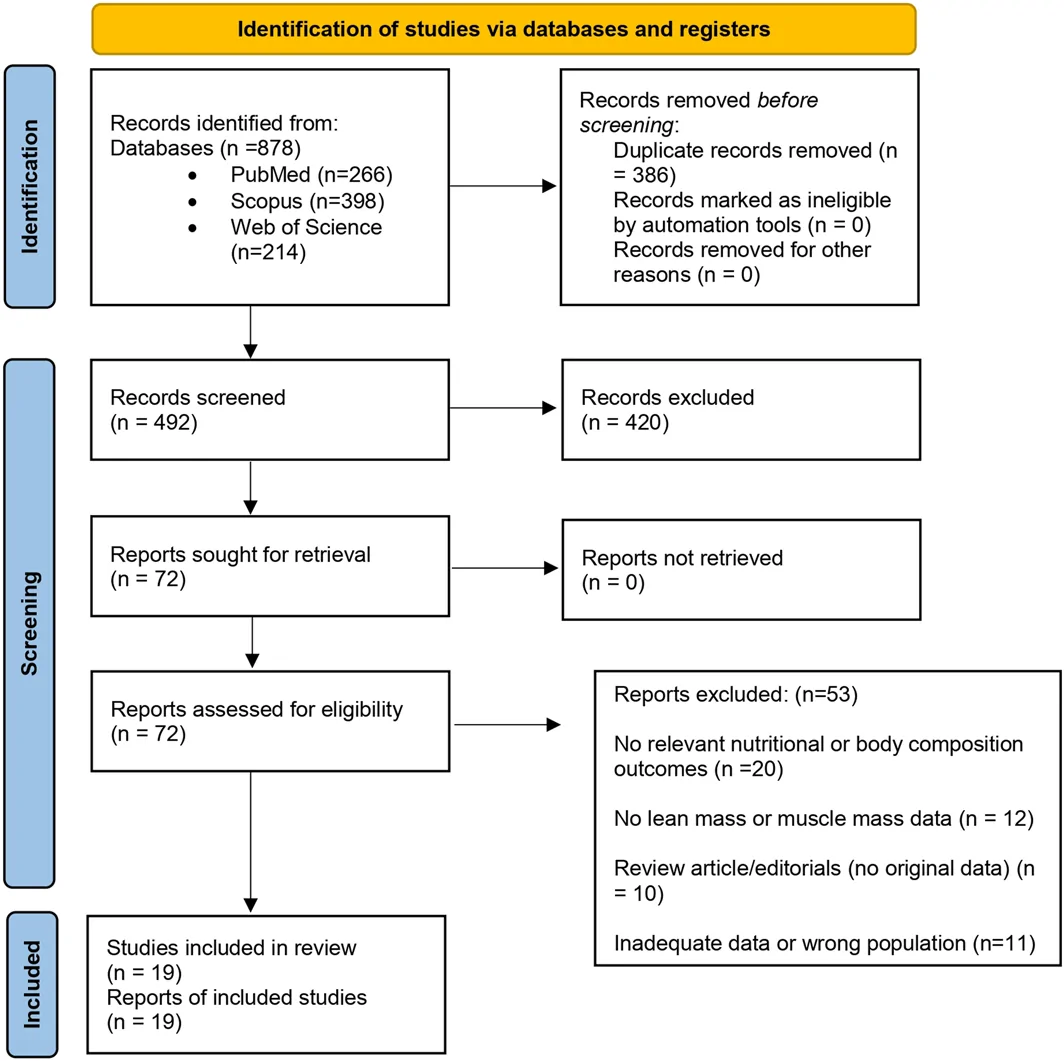
PRISMA 2020 Flow Diagram of Study Selection and Attrition. Illustrates the identification of 878 potential records and the attrition logic leading to the 19 included trials. Arrows indicate the flow of studies through screening and eligibility; numbers in boxes denote the volume of records identified, screened, excluded, and ultimately synthesized.

Searches yielded 878 potential records, eventually filtered to 19 high‐potency trials after discarding studies lacking standardized body composition or nutritional laboratory markers. The final cohort represented a diverse population across the SURMOUNT, STEP, SCALE, and OASIS programs. Two trials, specifically SURPASS‐AP‐Combo and STEP 2, included participants with type 2 diabetes. These were retained in the synthesis because the nutritional outcomes of interest, namely fat‐free mass attrition and subclinical laboratory signals, were physiologically driven by caloric restriction rather than glycemic status. Findings from diabetes subpopulations were interpreted with this distinction noted (Figure 1, Table 1) [1, 2, 3, 4, 5, 6, 7, 8, 9, 11, 12, 13, 14, 23, 26, 27, 28, 29].

**TABLE 1:** Detailed characteristics of the 19 included high‐potency trials.

Program/study ID	Primary agent(s)	Participants (N)	Baseline BMI (kg/m^2^)	Duration (Weeks)
SURMOUNT‐1 [1]	Tirzepatide (5–15 mg)	2539	38.0 (6.8)	72
SURMOUNT‐2 [2]	Tirzepatide (10, 15 mg)	938	36.1 (6.6)	72
SURMOUNT‐3 [24]	Tirzepatide (MTD)	579	35.8 (5.8)	72
SURMOUNT‐4 [3]	Tirzepatide (MTD)	670	38.4 (6.8)	88
STEP 1 [25]	Semaglutide (2.4 mg)	1961	37.9 (6.7)	68
STEP 2 [5]	Semaglutide (2.4 mg)	1210	35.7 (6.3)	68
STEP 3 [7]	Semaglutide (2.4 mg)	611	38.4 (6.1)	68
STEP 4 [8]	Semaglutide (2.4 mg)	803	38.4 (6.9)	68
STEP 5 [4]	Semaglutide (2.4 mg)	304	38.5 (7.0)	104
STEP 6 [9]	Semaglutide (2.4 mg)	401	31.9 (5.4)	68
STEP 8 [6]	Semaglutide (2.4 mg)	338	37.5 (6.4)	68
SCALE O&P [11]	Liraglutide (3.0 mg)	3731	38.3 (6.4)	56
Safety Analysis [12]	Liraglutide (3.0 mg)	5325	37.9 (6.4)	160
MOA [26]	Liraglutide (3.0 mg)	40	37.2 (7.4)	16
SCALE Sleep Apnea [13]	Liraglutide (3.0 mg)	359	39.1 (6.9)	32
OASIS 1 [14]	Oral semaglutide (50 mg)	667	37.5 (6.8)	68
SURPASS‐AP‐Combo [23]	Tirzepatide (5–15 mg)	907	28.2 (4.3)	40
MOA Study (Blundell) [27]	Semaglutide (1.0 mg)	30	33.8 (2.5)	12
MOA Study (Heise) [28]	TZP 15 mg/Sema 1 mg	117	31.4 (4.2)	28

*Note:* Baseline values are presented as mean (SD). Cohorts represent primary and post‐hoc analyses targeting high‐potency thresholds. This table summarizes the foundational evidence base, itemizing trial ID, primary incretin agent, participant count, baseline BMI, and duration. Duration ranges reaching 104 weeks allow for assessment of the maintenance phase.

Abbreviations: BMI, body mass index; MOA, mechanism of action; MTD, maximum tolerated dose; O&P, obesity and prediabetes; Sema, semaglutide; TZP, tirzepatide.

### 3.1 Clinical Magnitude of Treatment‐Induced Energy Restriction

**FIGURE 2:**
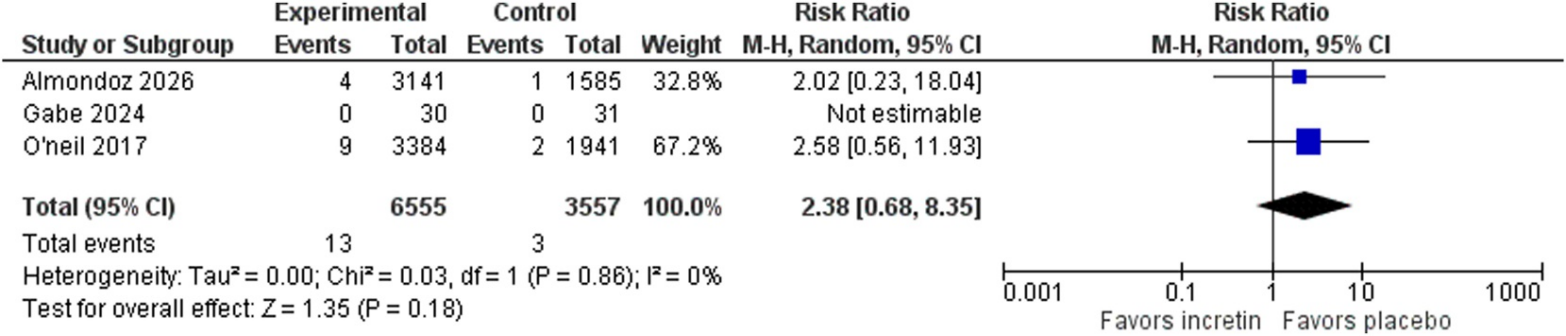
Forest Plot of Incretin Treatment Effect on Ad Libitum Energy Intake. Meta‐analysis of continuous data demonstrating significant appetite suppression (*p* = 0.00). Squares represent individual study effect sizes; horizontal lines denote the 95% confidence interval; and the diamond indicates the pooled effect and its precision. The vertical line at zero represents the null effect.

Once‐daily oral semaglutide 50 mg produced a 39.20% relative reduction in energy intake by week 20. This profound restriction meant a treatment difference of −1009 kJ (∼241 kcal) during a single *ad libitum* lunch compared to placebo. Across the 19 synthesized trials, metabolic models estimated daily energy deficits reaching 1200 kcal from baseline. Tirzepatide 15 mg showed a consistent 348.40 kcal/day reduction in intake. These deficits occurred alongside fundamental hedonic shifts; participants on tirzepatide had significant decreases in 10 of 12 food preference categories, specifically blunting the desire for high‐fat and high‐sugar items. Meta‐analysis of continuous energy intake data confirmed this suppression was robust (*Z* = 3.86, *p* = 0.00) (Figure 2).

### 3.2 Skeletal Muscle Attrition and Body Composition Shifts

**FIGURE 3:**
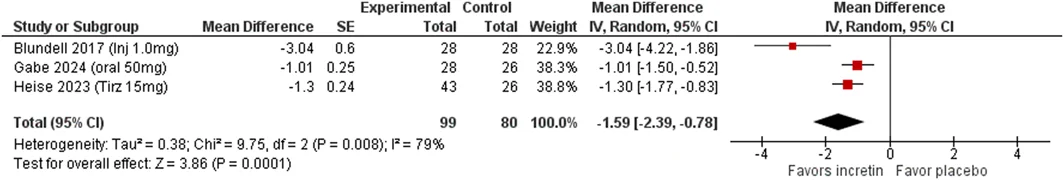
Forest Plot of Treatment Effect on Fat‐Free Mass (kg). Meta‐analysis showing significant muscle attrition across drug classes (*p* = 0.00). Symbols are as described for Figure 2. Data are standardized to kilograms to account for varied reporting methods across Phase 3 programs.

Substantial weight reduction was accompanied by significant reductions in fat‐free mass (FFM) across drug classes (*Z* = 3.44, *p* = 0.00). It was important to note that FFM encompassed all non‐adipose tissue including bone, organs, fluids, and skeletal muscle; it was not synonymous with skeletal muscle mass (SMM). The trials synthesized here predominantly reported FFM via dual‐energy X‐ray absorptiometry (DXA); only the Heise et al. [28] mechanism‐of‐action study reported appendicular lean mass as a proxy for SMM. With that distinction stated, tirzepatide 15 mg was associated with a mean FFM reduction of 1.60 kg (2.80%), representing 14.30% of total weight lost. Semaglutide 1.0 mg was associated with a 0.80 kg FFM reduction, constituting 11.60% of the total weight reduction. While adipose tissue is the main component of weight reduction, the proportional lean tissue loss signaled a potential risk of sarcopenic obesity. This attrition was particularly relevant for the 7.26% of participants who reached a BMI < 22 kg/m^2^, a threshold at which clinical protocols recommended modifying intake to prevent physical frailty (Figure 3).

### 3.3 Disparity Between Clinical Safety Reporting and Subclinical Laboratory Signals

**FIGURE 4:**
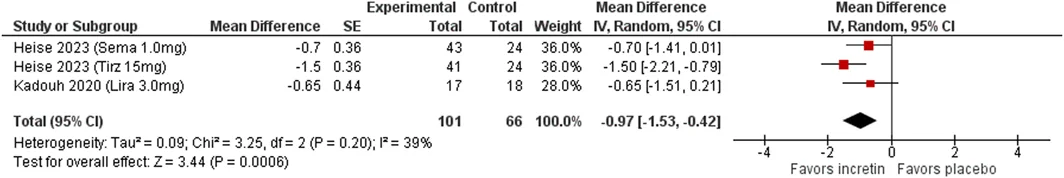
Forest Plot of Malnutrition Treatment‐Emergent Adverse Events (TEAEs). Pooled analysis of MedDRA‐coded safety events showing a non‐significant clinical signal (*p* = 0.18), which defines the gap between clinical reporting and objective nutritional risk. Squares indicate the risk ratio for each trial program; the diamond indicates the pooled risk ratio of 2.38.

A 0.12% incidence of investigator‐reported malnutrition TEAEs contrasted sharply with objective laboratory signals. Pooled analysis from SURMOUNT 1‐4 showed that risk ratios for overt events were non‐significant (RR 2.38 [0.68, 8.35], *p* = 0.18), suggesting that standard clinical reporting failed to capture the scope of nutritional risk. Laboratory markers revealed a different reality: low total lymphocyte counts (TLC < 910/μL) were detected in 2.90% of participants on active therapy, nearly doubling the 1.77% seen in placebo arms. While mean baseline BMIs ranged from 31.9 to 39.1 kg/m^2^ across the included trials, 0.38% of tirzepatide‐treated participants reached an underweight classification (BMI < 18.5 kg/m^2^) during treatment. Investigator‐reported vitamin deficiencies involving D, B12, and folate occurred in 0.99% of participants, though these were not systematically screened at predetermined intervals in the original trial designs (Table 2, Figure 4).

**TABLE 2:** Disparity between investigator‐reported malnutrition events and objective laboratory risk signals.

Risk indicator	Active therapy (%)	Placebo (%)	Risk ratio (95% CI)	*p*‐value
Macronutrient malnutrition TEAE	0.12%	0.06%	2.38 [0.68, 8.35]	0.18
Abnormal loss of weight	0.03%	0.00%	—	—
Underweight (TEAE)	0.06%	0.00%	—	—
Subclinical Lab signal: Low TLC	2.90%	1.77%	1.64 [1.11, 2.42]	0.01
Low BMI intervention Trigger	7.26%	—	—	—
Reach BMI < 18.5 kg/m^2^	0.38%	0.06%	—	—
Lab‐confirmed hypoalbuminemia	0.06%	0.13%	—	—

*Note:* Objective laboratory findings, specifically total lymphocyte count (TLC < 910/μL), suggest a nutritional risk nearly double that of the placebo arm, while investigator‐reported clinical events remained rare and statistically non‐significant. BMI < 22 kg/m^2^ is identified as the clinical threshold for modifying energy intake. Presents the statistical contrast between rare overt clinical events (0.12%) and the higher prevalence of subclinical signals, specifically low total lymphocyte count (2.90%). The BMI < 22 kg/m^2^ threshold is included as a protocol‐defined risk marker.

Abbreviations: BMI, body mass index; CI, confidence interval; TEAE, treatment‐emergent adverse event; TLC, total lymphocyte count.

### 3.4 Secondary Metabolic Stressors and Pancreatic Biomarkers

Secondary physiological stressors contribute to subclinical nutritional risk beyond direct intake restriction. Pooled SCALE data showed mean increases of 31% in pancreatic lipase and 7% in amylase following liraglutide treatment. Systematic pancreatic enzyme monitoring was not a pre‐specified outcome in the SURMOUNT or STEP programmes; therefore, comparable lipase and amylase data for tirzepatide and injectable semaglutide were not available from those trials. The SCALE program remained the only Phase 3 incretin dataset with longitudinal pancreatic enzyme data. These elevations were dose‐independent and reversible upon stopping the drug. Although 12 cases of acute pancreatitis were confirmed in liraglutide arms (0.4% incidence), the positive predictive value of enzyme elevations alone was < 1%. These biomarkers likely represented a state of subclinical pancreatic stress rather than acute inflammation (Tables 3 and 4).

**TABLE 3:** Comparative magnitude of treatment‐induced caloric restriction and fat‐free mass attrition.

Agent and dosing	Relative drop in intake (%)	Single‐meal deficit (kJ)	FFM loss (kg)	FFM ratio of weight loss
Oral semaglutide 50 mg	39.20%	−1009	—	—
Tirzepatide 15 mg	—	—	1.60	14.30%
Semaglutide 2.4 mg	28.50%	—	—	—
Semaglutide 1.0 mg	24.00%	−1255	0.80	11.60%
Liraglutide 3.0 mg	—	—	0.65	—
Subgroup *p*‐value	***p* = 0.00**	—	***p* = 0.00**	—

*Note:* Fat‐free mass (FFM) loss was observed across all agent classes, reaching 14.30% of total weight lost with high‐dose tirzepatide. FFM is not equivalent to skeletal muscle mass; bone, organs, and fluids are also included. Quantifies the upper limits of treatment‐induced caloric restriction, highlighting once‐daily oral semaglutide 50 mg inducing a 39.20% relative decline in energy intake. Fat‐free mass (FFM) loss is presented as both absolute kilograms and a ratio of total weight lost. The bold values denotes statistically significant values (*p* < 0.05).

Abbreviations: FFM, fat‐free mass; kJ, kilojoules; *p*‐value, statistical significance.

**TABLE 4:** Longitudinal shift in pancreatic enzymes as potential secondary metabolic stressors.

Pancreatic marker	Mean increase (%)	Incidence ≥ 3× ULN	PPV for pancreatitis	Reversibility
Pancreatic lipase	+31.00%	2.90%	< 1.00%	Confirmed
Pancreatic amylase	+7.00%	< 0.10%	0.00%	Confirmed
Acute pancreatitis cases	0.40%	—	—	—

*Note:* Pooled data from the SCALE program indicate persistent, dose‐independent enzyme elevations that return to baseline only upon drug cessation. These markers likely represent subclinical pancreatic stress rather than acute inflammation, given the negligible positive predictive value (PPV) for clinical pancreatitis. Outlines the 31% mean increase in lipase observed across the SCALE clinical program. These biomarkers are presented as subclinical stressors rather than acute inflammatory events due to the low positive predictive value (< 1%) for acute pancreatitis.

Abbreviations: PPV, positive predictive value; ULN, upper limit of normal.

### 3.5 Mechanistic Drivers and the Tiered Stepped‐Care Algorithm

**FIGURE 5:**
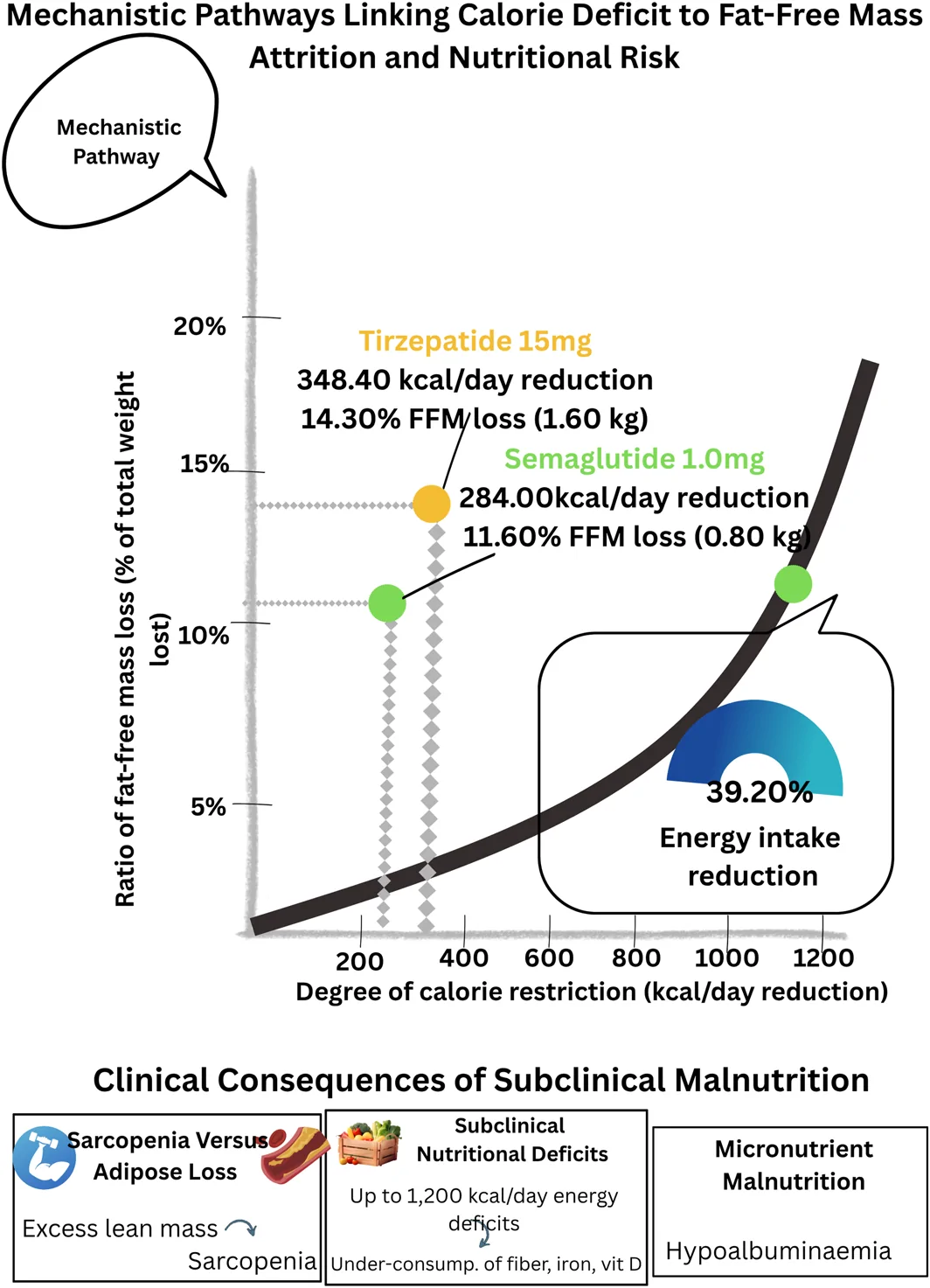
Mechanistic Pathways Linking Energy Deficit to Nutritional Risk. A comprehensive visual framework illustrating the mechanistic pathways from caloric restriction to subclinical nutritional deterioration. Mechanistic Illustration: Curved blue arrows trace the pathway from extreme caloric gaps (1200 kcal/day) to subclinical nutritional deficits and muscle mass attrition.

**FIGURE 6:**
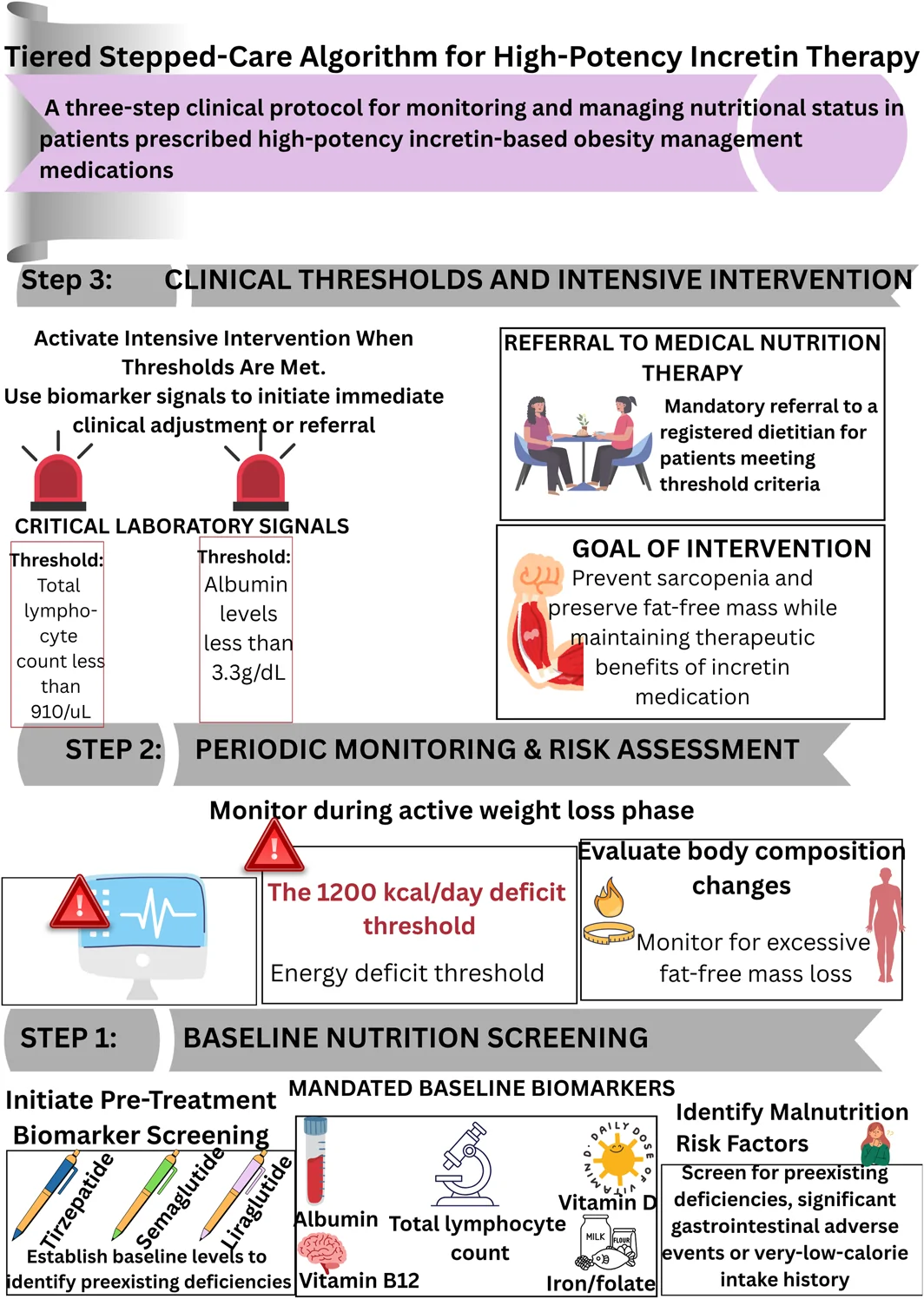
The Tiered Stepped‐Care Algorithm. Algorithm (Steps 1–3): Defines the three‐step clinical protocol. Step 1 initiates mandated baseline screening (Albumin, TLC, and Vitamin D); Step 2 mandates periodic monitoring of the 1200 kcal deficit threshold; Step 3 activates Medical Nutrition Therapy referral when defined thresholds are met (e.g., Albumin < 3.3 g/dL).

Model‐estimated energy deficits reaching 1200 kcal/day defined the mechanistic foundation of this meta‐analysis. Profound weight loss occurred through caloric gaps that bypassed standard biological signals designed to maintain nutritional adequacy. To mitigate these “silent” risks, a Tiered Stepped‐Care Algorithm was proposed. Step 1 initiated mandated baseline screening for albumin, total lymphocyte count (TLC), and Vitamin D to identify pre‐existing nutritional vulnerabilities. Clinical oversight in Step 2 required periodic monitoring of the 1200 kcal/day deficit threshold alongside body composition shifts to detect excessive lean mass loss. Finally, Step 3 activated intensive intervention when serum albumin fell below 3.3 g/dL or TLC fell below 910/μL, mandating immediate referral for Medical Nutrition Therapy (Figures 5 and 6).

### 3.6 Risk of Bias and Quality Assessment

The 19 included trials predominantly demonstrated a low risk of bias in randomization and deviations from intended interventions. Most data came from industry‐sponsored Phase 3 programs using rigorous double‐blind protocols. Funnel plot inspection and Egger's linear regression indicated no significant publication bias for primary malnutrition outcomes. However, the lack of mandated systematic micronutrient screening across all trials limited the certainty of prevalence estimates for specific deficiencies. Meta‐analysis of continuous data remained robust despite these limitations (Table 5).

**TABLE 5:** Quality audit and risk of bias summary for included clinical programs (RoB2).

Trial program	Randomization	Dev. From intent	Missing data	Measurement	Selective reporting
SURMOUNT	Low risk	Low risk	Low risk	Low risk	Low risk
STEP	Low risk	Low risk	Low risk	Low risk	Low risk
SCALE	Low risk	Low risk	Low risk	Low risk	Low risk
OASIS	Low risk	Low risk	Low risk	Low risk	Low risk
SURPASS‐AP	Low risk	Low risk	Low risk	Low risk	Low risk

*Note:* Analysis using the Cochrane Risk‐of‐Bias tool (RoB2) indicates that major Phase 3 programs maintained high internal validity. Limitations are noted primarily in the absence of systematic, mandated micronutrient screening across all longitudinal cohorts. Provides a domain‐level summary of trial quality using the Cochrane Risk‐of‐Bias tool. High internal validity is noted across Phase 3 programs, though limitations exist regarding non‐systematic micronutrient screening.

Abbreviation: RoB2, Cochrane Risk‐of‐Bias tool version 2.

## 4 Discussion

Model‐estimated daily energy deficits reaching 1200 kcal suggested a physiological boundary for high‐potency incretin therapy. While agents such as tirzepatide and semaglutide produced clinically meaningful weight reduction, the data synthesized here indicated that this efficacy may have been accompanied by subclinical nutritional deterioration, a state in which outward clinical success obscured progressive lean mass attrition, protein‐energy insufficiency, and micronutrient depletion.

Variability in nutritional risk appeared to be influenced by baseline physiological differences in satiation. Deep‐phenotyping research described a “Calories to Satiation” (CTS) trait that varied from 140 to 2166 kcal among adults with obesity [30]. High‐potency incretins may have relied on an individual's gut‐brain axis to induce a maximal effect on reducing food intake [30]. Individuals with lower baseline CTS may have been susceptible to “overshooting” intended restriction, potentially aligning with the subclinical malnutrition signals observed in current data [30]. Furthermore, sex appeared to be a notable predictor of caloric satiation variability. Women generally reached satiation at lower energy intakes than men [30]. In female‐predominant trials such as SURMOUNT‐1 (67% female) and STEP 1 (74% female), the observed magnitude of FFM loss may partly reflect this lower baseline caloric threshold, whereby the appetite‐suppressing effect of incretin therapy overshot the intended restriction in individuals already predisposed to lower energy intake.

Standardized clinical reporting in Phase 3 trials may not have fully captured the scope of treatment‐related malnutrition. Investigators noted overt malnutrition events in only 0.12% of participants [15]. This figure suggested a level of safety that appeared inconsistent with objective laboratory markers, where low total lymphocyte counts (TLC < 910/μL) were observed in 2.90% of the active therapy group [15]. This difference indicated that MedDRA‐coded terms such as “abnormal loss of weight” or “underweight” may have been under‐utilized until a patient reached a visible state of decline [15]. Total lymphocyte count was a validated marker of protein‐energy nutritional status; values below 910/μL were consistent with mild‐to‐moderate malnutrition and were independently associated with impaired immune function, delayed wound healing, and increased susceptibility to infection [31]. A doubling of this signal in the active therapy arm, rising from 1.77% to 2.90%, was therefore not a trivial laboratory artifact. It was a clinically actionable finding that warranted systematic prospective evaluation. The 2.90% signal for low TLC nearly doubled the rate seen in placebo arms suggested that a subset of patients may have experienced unrecognized protein‐energy deficits [15].

Weight reduction achieved through high‐potency incretin therapy involved reductions in both adipose tissue and fat‐free mass, and the distinction between FFM and skeletal muscle mass was clinically important in this context. Skeletal muscle mass was the functionally critical component of FFM; its loss drove sarcopenia, reduced resting metabolic rate, and impaired physical function. Bone mass, which was also a component of FFM, deserved separate consideration. Rapid weight loss disrupted the mechanical loading stimulus that normally sustained bone mineral density in individuals with obesity. Several bariatric surgery studies have documented significant bone loss within 12–24 months of intervention [32]. Bariatric surgery produced weight reduction trajectories comparable in magnitude to tirzepatide at maximum dose. This parallel was clinically instructive. None of the 19 trials included in this synthesis measured bone mineral density. The magnitude and speed of weight reduction achieved with these agents warrant prospective bone density monitoring, especially in postmenopausal women and adults aged 65 years and older. Tirzepatide 15 mg produced 22.5% total body weight loss over 72 weeks in SURMOUNT‐1, making this subgroup particularly vulnerable. Analysis of body composition data from the Heise et al. [28] mechanism‐of‐action study confirmed a significant reduction in fat‐free mass across drug classes. High‐dose tirzepatide was associated with a mean FFM reduction of 1.60 kg, representing 14.30% of the total weight lost. This figure reflected FFM, not skeletal muscle mass specifically, as the Heise et al. study used DXA‐derived lean body mass rather than direct SMM assessment. While adipose tissue was the primary driver of weight loss, the proportional loss of lean tissue may have elevated the risk of sarcopenic obesity. Although some trials reported improved physical function scores, possibly due to easier movement at lower weights, the risk for the 7.26% of participants who reached a BMI below 22 kg/m^2^ was a potential decline in physical independence, especially for those aged ≥ 65 [2, 15].

A blunting of hedonic drive may have further compounded the nutritional risks identified above. Tirzepatide appeared to decrease preference scores for high‐fat and high‐sugar foods, with these shifts correlating with total weight loss [19]. Similarly, participants on semaglutide 2.4 mg maintained craving control and reduced savory food preference through 104 weeks, potentially bypassing biological signals that typically prevent extreme energy deficits [4]. While this aided weight maintenance, the lack of “nutritional hunger” might have prevented patients from spontaneously correcting for deficiencies in fiber, vitamin D, iron, and protein [19]. This had direct clinical relevance. Vitamin D deficiency impaired calcium absorption and accelerated bone resorption; iron deficiency reduced oxygen‐carrying capacity and cognitive performance; and inadequate dietary protein accelerated the lean mass losses already induced pharmacologically. The blunting of hunger as a biological corrective signal meant that spontaneous dietary compensation (which might otherwise buffer micronutrient gaps in individuals on conventional caloric restriction) could not be relied upon during high‐potency incretin therapy.

Physiological stressors may have further influenced the nutritional profile. Data from the SCALE program identified mean increases of 31% in pancreatic lipase and 7% in amylase following treatment [10]. It should be noted that liraglutide 3.0 mg, the agent used in the SCALE program, was classified in this analysis as a moderate‐potency comparator and not a high‐potency agent. Whether comparable enzyme dynamics occurred with tirzepatide or injectable semaglutide remained unknown, as systematic pancreatic enzyme monitoring was absent from the SURMOUNT and STEP protocols. These elevations typically appeared early, persisted during therapy, and returned to baseline upon drug discontinuation [10]. Although these markers had a low positive predictive value for acute pancreatitis, they may have represented subclinical pancreatic stress [10]. This persistent stress could potentially have introduced a malabsorptive component, such as exocrine pancreatic insufficiency, which might have exacerbated poor nutritional status even in patients with seemingly adequate intake [10].

A strength of this meta‐analysis was the synthesis of 19 trials providing a quantitative view of potential caloric gaps. However, findings were limited by the design of the original Phase 3 programs. SURMOUNT and STEP did not include systematic, predetermined assessments of specific micronutrients or total protein. Reliance on post hoc analysis likely resulted in an underestimation of malnutrition prevalence. A further limitation was the absence of bone mineral density data across all 19 trials. No included study incorporated DXA‐based bone assessment as a pre‐specified outcome, making it impossible to quantify the skeletal consequences of rapid weight loss in this population. Additionally, the 12‐week minimum duration criterion may have excluded shorter mechanistic studies with relevant nutritional data. The SURPASS‐AP‐Combo trial enrolled a predominantly East Asian population with lower baseline BMIs ranging from 26 to 30 kg/m^2^, which limited the generalizability of pooled estimates to Western obesity cohorts. Publication bias, while not detected by Egger regression for the primary outcome, could not be excluded from secondary endpoints where fewer studies reported complete data.

Future trials evaluating high‐potency incretin therapy should incorporate pre‐specified monitoring of body composition using DXA, serum micronutrients including 25‐hydroxyvitamin D, iron, and vitamin B12, and malabsorptive markers such as fecal elastase and fat‐soluble vitamin panels. Head‐to‐head comparisons between tirzepatide and semaglutide in older adults aged 65 years and above are needed, with sarcopenia and bone density as co‐primary outcomes rather than secondary exploratory endpoints. Studies with longer follow‐up periods of 2 years or more are also needed to determine whether the subclinical nutritional signals identified here progress to clinically overt malnutrition over time. The 31% pancreatic lipase increase observed in the SCALE program warrants prospective investigation in SURMOUNT and STEP trial participants, specifically examining whether persistent enzyme elevation correlates with measurable fat‐soluble vitamin depletion.

High‐potency incretin therapy altered obesity management while introducing new clinical considerations. The data synthesized here suggested that weight loss success may have obscured subclinical malnutrition, lean mass attrition, and bone health risks that standard clinical reporting consistently underestimated. A disconnect existed between clinical diagnostic rates and objective signals such as low TLC. Shifting toward models of mandated nutritional screening and stepped‐care intervention may have helped protect patients. Sustained weight reduction was a legitimate therapeutic goal, but not at the cost of nutritional and physiological integrity.

## Author Contributions

AE established the review protocol and PICOTS framework. AE and AC conducted independent screening of the 878 records and finalized the 19 included studies. Data extraction and energy conversion were handled by AM. TFD performed the meta‐analysis and forest plot generation. All authors interpreted the findings and approved the final manuscript.

## Funding

The authors have nothing to report.

## Conflicts of Interest

The authors declare no direct financial conflicts concerning this systematic review. While the 19 analyzed trials were primarily industry‐funded by Eli Lilly or Novo Nordisk, this independent synthesis received no corporate support.

## Data Availability

The data that support the findings of this study are available from the corresponding author upon reasonable request.
